# Role of Rheum Polysaccharide in the Cytokines Produced by Peripheral Blood Monocytes in TLR4 Mediated HLA-B27 Associated AAU

**DOI:** 10.1155/2013/431232

**Published:** 2013-09-16

**Authors:** Xuhui Liu, Xiaofeng Hu, Xiaosheng Zhang, Zhongqiu Li, Hong Lu

**Affiliations:** Department of Ophthalmology, Beijing Chaoyang Hospital, Capital Medical University, No. 8 Baijiazhuang Road Chaoyang District, Beijing 100020, China

## Abstract

*Purpose*. To evaluate the effect of a traditional Chinese medicine, Rheum Polysaccharide (RP), on the in vitro production of tumor necrosis factor alpha (TNF-**α**) and interleukin-10 (IL-10) by lipopolysaccharide- (LPS-)stimulated human monocytes from HLA-B27 associated acute anterior uveitis patients of convalescence stage. *Method*. PBMC samples were isolated from 10 HLA-B27 associated acute anterior uveitis, incubated, respectively, and divided into 4 groups as follows: (1) controls, PBS was added in final concentration of 1 mg*·*L^−1^, (2) stimulated by LPS, LPS was added in final concentration of 1 mg*·*L^−1^, (3) stimulated by LPS + HTA125, 30 minutes before the adding of LPS in final concentration of 1 mg*·*L^−1^, the final concentration of 5 mg*·*L^−1^ of the HTA125 was added, and (4) stimulated by LPS + RP, 30 minutes before the adding of LPS in final concentration 1 mg*·*L^−1^, the final concentration 100 mg*·*L^−1^ of the RP was added. Supernatants were used to quantify the amounts of TNF-**α** and IL-10 released in time course using enzyme-linked immunosorbent assay (ELISA). *Result*. After stimulated by lps, the concentrations of TNF-**α** and IL-10 in culture supernatants of patients are significantly higher than control group at all time points (*P* < 0.01). Blockage of TLR-4 by HTA125 can decrease the production of TNF-**α** and IL-10 compared with lps group (*P* < 0.01, except at 4 h group of IL-10). Concentration of TNF-**α** and IL-10 also decreases in the LPS + RP group (*P* < 0.01) but not so significantly as in the LPS + HTA125 group. *Conclusion*. As anti-TLR4 monoclonal antibodies, rheum Polysaccharide can also inhibit the secretion of cytokines produced by monocytes from HLA-B27 positive AAU patients of convalescence stage.

## 1. Introduction

In recent years, a large number of studies have shown that Rheum polysaccharide is the major immune active substance in a plenty of traditional Chinese medicine, which can be immune promoters and regulators. Rheum is Chinese traditional and commonly used herbs and has a long history of medicinal application. Rheum polysaccharide has been demonstrated to be resistant to CD4 T cells expansion and modulates Th1 and Th2 cytokines production [1, 2]. HLA-B27 associated acute anterior uveitis (AAU) is the most common form of uveitis, accounting for 18%–32% of all cases. We have previously reported that LPS-Toll like receptor 4 (TLR4) signal transduction was potentially associated with the pathogenesis of EIU [3]. 

TLRs are pattern recognition receptors (PRR) that recognize “signature patterns” of microbes, called pathogen-associated molecular patterns (PAMPs). PAMPs are highly conserved molecules shared by many microorganisms of a particular class, such as LPS in gram-negative bacteria which can respond to TLR4. TLR4 is absolutely essential for inflammatory responses to LPS. In the animal model for AAU, the C3H/HeN strain of mice, which are highly sensitive to develop EIU, has normal functional TLR4, while the congenic C3H/HeJ strain with nonfunctional TLR4 does not develop EIU [4]. 

In the present study, we evaluated the protective effect of RP and compared it with anti-TLR4 monoclonal antibodies (HTA125) on monocytes stimulated by LPS in HLA-B27 associate AAU. We investigated the role of RP in inflammation and immune response by measuring the time course of changes in concentrations of cytokine secretion (TNF-*α* and IL-10) by monocytes isolated from HLA-B27 associated AAU. 

## 2. Materials and Methods

### 2.1. Study Population

Patients with HLA-B27 associate AAU were recruited from the Eye Clinic of Beijing Chao-Yang Hospital, from June 2011 to September 2011, referring the International Uveitis Study Group standard for diagnosis and classification [5]. Ten subjects who fulfilled the following inclusion/exclusion criteria were invited to participate in the study. All eligible subjects have written informed consent, and the ethics committee of Capital Medical University has approved this clinical research protocol. The patients aged 24–42 yrs, with an average of 33 yrs. All the patients were given topical corticosteroid, NSAIDS, and mydriatic treatment after diagnosis: 10 g·L^−1^ fluorometholone, qid—6 times a day, diclofenac sodium eye solution, qid, and tropicamide, bid. One month later, the patients recovered, and all the symptoms and signs disappeared.

### 2.2. Experimental Reagents

The reagents used are lipopolysaccharide (*V. cholera*, classical biotype, serotype Ogawa Lanzhou, China). Rheum polysaccharide (from Lanzhou University Pharmacology Lab, purity 90%). Ficoll-Hypaque solution (HAO YANG biological manufacture, Tianjin, China). RPMI medium-1640 and PBS (Gibco, NY, USA). HTA125 (Abcam UK). TNF-*α*, IL-10 ELISA kits (Quantikine; RD Systems, Abingdon, UK).

### 2.3. Monocytes Isolation

About 20 mL of venous blood was collected from each subject by venipuncture into heparinized tubes and immediately processed. The blood was diluted 1 : 1 in saline solution and layered over Ficoll-Hypaque solution for separation of mononuclear cells. Recovered peripheral blood mononuclear cells (PBMC) were washed three times with D-PBS and resuspended in RPMI medium-1640 (Gibco, NY, USA) with heat inactivated 10% fetal bovine serum, L-glutamine (2 mM), penicillin (100 U/mL), and streptomycin (1%) and adjusted to a concentration of 1 × 10^6^ cells/mL using a hemocytometer. Cell viability was ≥95% which checked with trypan blue staining under the microscope. Aliquots of each cell suspension were then plated into chambers of 24-well culture dishes. The plates were maintained in a humidified incubator with 5% CO_2_ at 37°C for 4 h to allow the monocytes to adhere to the plates. Nonadherent cells were removed by washing with Hank's balanced salt solution (HBSS). 

### 2.4. Experimental Design

Monocyte samples were isolated from 10 HLA-B27 associated AAU, divided into 4 groups as follows: (1) controls, PBS was added in final concentration of 1 mg·L^−1^, (2) stimulated by LPS (*V. cholera*, classical biotype, serotype Ogawa), LPS was added in final concentration of 1 mg·L^−1^, (3) stimulated by LPS + HTA125 (Abcam UK), 30 minutes before the adding of LPS in final concentration 1 mg·L^−1^, the final concentration 5 mg·L^−1^ of the HTA125 was added, and (4) stimulated by LPS + RP, 30 minutes before the adding of LPS in final concentration 1 mg·L^−1^, the final concentration 200 mg·L^−1^ of the RP was added. Samples of culture medium were harvested at different times (4 h, 8 h, 12 h and 24 h) after stimulation by LPS.

### 2.5. Sample Storage and Outcome Measures

Cell culture supernatants were harvested and stored in microtubes at −80°C, and TNF-*α*, IL-10 assays were performed on freshly thawed samples, with a standard quantifiable sandwich enzyme immunoassay technique. ELISAs were performed according to the manufacturer's protocol. The measurements were performed using an automated enzyme-linked immunosorbent assay reader (Wellscan MK3, Finland) at an optic absorbance value of 450 nm.

### 2.6. Statistical Analyses

The means (±standard deviations) of the data obtained were calculated. The statistical analysis was carried out using the Statistical Package for Social Sciences version 13 (SPSS Inc, Chicago, IL). Analysis of variance was carried out for multiple comparisons using the paired samples *t*-test. A *P* value <0.05 was accepted as being statistically significant.

## 3. Result 

**Figure 1:**
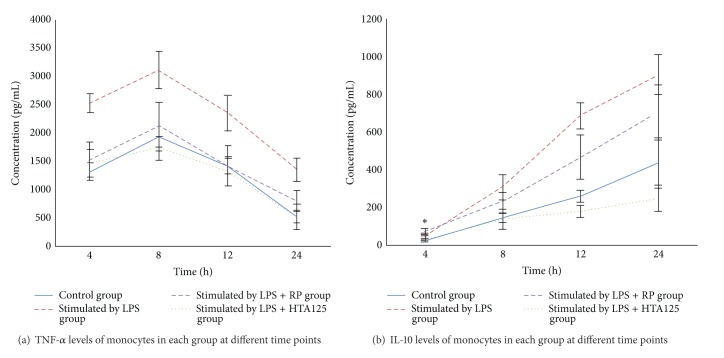
TNF-*α* (a) and IL-10 (b) levels in a time-dependent manner. Peripheral blood monocytes (1 × 10^6^ cells/mL) were pretreated for indicated time with 5 mg·L^−1^ HTA-125 or 100 mg·L^-1 ^RP and then incubated with 1 mg·L^−1^ LPS for another 24 h. Cell-free supernatants were collected. The relative concentrations of TNF-*α* and IL-10 in the supernatants were determined by quantitative ELISA assay. Cytokine levels were expressed as mean ± SD in pg/mL. Paired samples *t*-test was used to examine the differences. *P > 0.05 compared with LPS of IL-10.

The concentrations (pg/mL) of TNF-*α* and IL-10 in the supernatants of the stimulated monocytes are shown in Figures 1(a) and 1(b). The ability of HTA-125 or RP to inhibit the production of inflammatory cytokines was tested in LPS stimulated monocytes.

After stimulation by LPS, the concentrations of TNF-*α* (reaching 3115.84 pg/mL within 8 hours) and IL-10 (reaching 905.73 pg/mL within 24 hours) in culture supernatants of monocytes are significantly higher than control group (the concentrations of TNF-*α* reaching 1929.76 pg/mL within 8 hours and IL-10 reaching 435.98 pg/mL within 24 hours) at all-time points (*P* < 0.01). Blockage of TLR-4 by HTA125 can decrease the production of TNF-*α* (reaching 1738.01 pg/mL within 8 hours) and IL-10 (reaching 249.39 pg/mL within 24 hours) compared with lps group (*P* < 0.01, except at 4 h group of IL-10). Concentrations of TNF-*α* (reaching 2117.30 pg/mL within 8 hours) and IL-10 (reaching 702.43 pg/mL within 24 hours) also decrease in the LPS + RP group (*P* < 0.01) but not so significantly as in the LPS + HTA125 group. In all groups, the concentration of TNF-*α* reaches the peak within 8 hours after the start of incubation and then tends to decrease. Release of IL-10 starts to increase after the start of incubation and reaches the maximum within 24 hours.

## 4. Discussion 

HLA-B27-associated acute anterior uveitis can cause visual impairment and blindness with a high incidence of recurrence and a mean duration of each episode of 4–6 weeks. DEX is one of the most widely used drugs for treatment of AAU in clinic; however, severe systemic and ocular side effects limit its use, particularly for long term therapy [6]. Preclinical and clinical studies have demonstrated that Rheum polysaccharides exhibited numerous beneficial therapeutic properties, including immunostimulation, antiinfection, antitumor, and other therapeutic aspects [7–9]. In this paper, we evaluated the protective effect of RP, a kind of polysaccharide extracted from Rheum, on monocytes from HLA-B27 associated AAU patients induced by LPS, and compared its efficacy with HTA125.

TLR4 expression has been demonstrated in macrophages, peripheral blood monocytes, dendritic cells (DCs), and various tissues [10, 11]. Among the earliest phagocytes to respond to infection are tissue macrophages, which originate as monocytes in the peripheral blood [12]. The activation of TLR4 + macrophages by LPS induces various proinflammatory cytokines, chemokines, and antimicrobial activities. Therefore, macrophages play a key role in the pathogenesis of EIU, as these innate immune cells are expected to be able to respond rapidly to LPS from Gram-negative bacteria [13]. In our previous research, We discovered that the concentration of TNF-*α* and IL-10 excreted by PBMCs from HLA-B27 positive patients was higher than normal controls, and cytokine levels from HLA-b27 patients' had significantly higher rises than normal people after LPS stimulation. So, in this study, we choose monocytes from HLA-B27 positive AAU patients peripheral blood and HTA125—TLR4 blocker to investigate the effect of RP. 

The ability of macrophage to secrete cytokine is critical to amplify and orientate the immune response. We assessed the secretion of TNF-*α* and IL-10 by macrophages. Tumor necrosis factor-*α* is a cytokine involved in systemic inflammation and is a member of a group of cytokines that stimulate the acute phase reaction. It is produced chiefly by activated macrophages and can regulate other immune cells. Pérez-Guijo et al. [14] and Santos Lacomba et al. [15] observed the increased level of TNF-*α* in the serum and aqueous humor of AAU patients and the elevated level in the serum of patients with recurrence. Interleukin 10 (IL-10) is an anti-inflammatory cytokine that is primarily produced by monocytes. Muzio et al. [16] found that IL-l0 can downregulate the expression of TLR4 in LPS-mediated signaling pathway. Calder et al. [17] observed the decrease of IL-10 concentration in aqueous humor of uveitis patients. In this experiment, the cells were incubated with LPS and RP, HTA-125 alone and in combination, and the concentration of TNF-*α* reaches the peak within 8 hours after the start of incubation after stimulated by LPS. Release of IL-10 starts to increase after the start of incubation and reaches the maximum within 24 hours. Blockage of TLR-4 by HTA125 can decrease the production of TNF-*α* and IL-10 compared with lps group. RP also significantly decreased TNF-*α* and IL-10 secretion by monocytes but less effectively than HTA-125.

In our study, RP exhibited protective effect in reducing LPS-induced inflammatory reaction but not so remarkable as HTA125. The possible mechanism may be different blockage of signalling pathways of HTA 125 and RP. HTA125 is the specific monoclonal antibody of TLR-4. RP contains 49% mannose which could potentially bind to MR. Polysaccharides with multiple repeats may engage multiple ligands, resulting in a super cross-linking of the receptors and blocking or engulfing the endocytosis process [18]. RP may affect the secretion of cytokine through targeting MR and MR-mediated ligand binding and endocytosis of macrophages. Study by Liu et al. [1] has demonstrated that LAM(a natural ligand of MR and strong agonist) or RP alone significantly increased the secretion of IFN-*γ* by macrophages in vitro and LAM was more potent than RP. Interestingly, the elevation of secretion of IFN-*γ* was diminished when LAM and RP were incubated together with macrophages, suggesting that RP may have the similar characteristic of partial agonist.

## 5. Conclusion

RP can inhibit the secretion of cytokines produced by monocytes stimulated with LPS. But the mechanism is more complex than HTA125. RP could be a potential novel therapeutic agent for treatment of patients with HLA-B27 associated AAU. However, this concept needs to be verified in vivo and clinically. 

## References

[1] Liu L, Wang Z-P, Xu C-T (2003). Effects of Rheum tanguticum polysaccharide on TNBS-induced colitis and CD4^+^T cells in rats. *World Journal of Gastroenterology*.

[2] Liu L, Mei QB, Wang ZP, Zhou YM (2005). The effects of Rheum tanguticum polysaccharide on polarization of Th1 and Th2 cells in TNBS-induced colitis in murine. *International Journal of Biological Sciences*.

[3] Chen W, Hu X, Zhao L, Li S, Lu H (2009). Expression of toll-like receptor 4 in uvea-resident tissue macrophages during endotoxin-induced uveitis. *Molecular Vision*.

[4] Xu Y, Chen W, Lu H (2010). The expression of cytokines in the aqueous humor and serum during endotoxin-induced uveitis in C3H/Hen mice. *Molecular Vision*.

[5] Jabs DA (2005). Standardization of uveitis nomenclature for reporting clinical data. Results of the first international workshop. *American Journal of Ophthalmology*.

[6] Xu CT, Pan BR (1999). Current medical therapy for ulcerative colitis. *World Journal of Gastroenterology*.

[7] Schepetkin IA, Quinn MT (2006). Botanical polysaccharides: macrophage immunomodulation and therapeutic potential. *International Immunopharmacology*.

[8] Tzianabos AO (2000). Polysaccharide immunomodulators as therapeutic agents: structural aspects and biologic function. *Clinical Microbiology Reviews*.

[9] Sergeev AV, Revazova ES, Denisova SI, Kalatskaia OV, Rytenko AN (1985). Immunomodulating and antitumor activity of polysaccharides of plant orgin. *Bulletin of Experimental Biology and Medicine*.

[10] Zarember KA, Godowski PJ (2002). Tissue expression of human Toll-like receptors and differential regulation of Toll-like receptor mRNAs in leukocytes in response to microbes, their products, and cytokines. *Journal of Immunology*.

[11] Mita Y, Dobashi K, Endou K (2002). Toll-like receptor 4 surface expression on human monocytes and B cells is modulated by IL-2 and IL-4. *Immunology Letters*.

[12] Parker LC, Prince LR, Sabroe I (2007). Translational mini-review series on Toll-like receptors: networks regulated by Toll-like receptors mediate innate and adaptive immunity. *Clinical and Experimental Immunology*.

[13] Pouvreau I, Zech J-C, Thillaye-Goldenberg B, Naud M-C, Van Rooijen N, De Kozak Y (1998). Effect of macrophage depletion by liposomes containing dichloromethylene-diphosphonate on endotoxin-induced uveitis. *Journal of Neuroimmunology*.

[14] Pérez-Guijo V, Santos-Lacomba M, Sánchez-Hernández M, Castro-Villegas MDC, Gallardo-Galera JM, Collantes-Estévez E (2004). Tumour necrosis factor-alpha levels in aqueous humour and serum from patients with uveitis: the involvement of HLA-B27. *Current Medical Research and Opinion*.

[15] Santos Lacomba M, Marcos Martín C, Gallardo Galera JM (2001). Aqueous humor and serum tumor necrosis factor-*α* in clinical uveitis. *Ophthalmic Research*.

[16] Muzio M, Bosisio D, Polentarutti N (2000). Differential expression and regulation of toll-like receptors (TLR) in human leukocytes: selective expression of TLR3 in dendritic cells. *Journal of Immunology*.

[17] Calder VL, Shaer B, Muhaya M (1999). Increased CD4+ expression and decreased IL-10 in the anterior chamber in idiopathic uveitis. *Investigative Ophthalmology and Visual Science*.

[18] Chieppa M, Bianchi G, Doni A (2003). Cross-linking of the mannose receptor on monocyte-derived dendritic cells activates an anti-inflammatory immunosuppressive program. *Journal of Immunology*.

